# Why China is important in advancing the field of primatology

**DOI:** 10.24272/j.issn.2095-8137.2018.012

**Published:** 2018-05-12

**Authors:** Paul A. Garber

**Affiliations:** Department of Anthropology, Program in Ecology, Evolution, and Conservation Biology, University of Illinois, Urbana Illinois 61801, USA

**Keywords:** China, Conservation, Primate research, Ecology

## Abstract

Over the past few decades, field studies conducted by Chinese primatologists have contributed significant new theoretical and empirical insights into the behavior, ecology, biology, genetics, and conservation of lorises, macaques, langurs, snub-nosed monkeys, and gibbons. With the recent establishment and inaugural meeting of the China Primatological Society in 2017, China has emerged as a leading nation in primate research. Several research teams have conducted long-term studies despite the difficult challenges of habituating and observing wild primates inhabiting mountainous temperate forests, and the fact that some 80% of China’s 25–27 primate species are considered vulnerable, endangered, or critically endangered and are distributed in small isolated subpopulations. In going forward, it is recommended that primatologists in China increase their focus on seasonal differences in the social, ecological, physiological, and nutritional challenges primates face in exploiting high altitude and cold temperate forests. In addition, provisioning as a habitation tool should be minimized or eliminated, as it is difficult to control for its effects on group dynamics, patterns of habitat utilization, and feeding ecology. Finally in the next decade, Chinese primatologists should consider expanding the taxonomic diversity of species studied by conducting research in other parts of Asia, Africa, and the Neotropics.

## INTRODUCTION

Since the first study of a primate in the wild by Clarence Raymond Carpenter in 1931, research in field primatology has been conducted principally by scientists from the United States, Europe, and Japan; regions largely devoid of nonhuman primates (Sussman, 2010). Over the past 20 years this has begun to change, with primate habitat countries such as Brazil, Mexico, Argentina, India, and China developing a cadre of highly-trained researchers and a national primatological society (http://www.internationalprimatologicalsociety.org/affiliatedsocieties.cfm). Among this group, China stands out for its rapid economic development, the government’s commitment to funding high-quality scientific research, within-country access to state-of-the-art genetic, hormonal, imaging, geographic information system (GIS), and nutritional laboratories, as well as strong expertise in computer modeling and statistical analysis (King, 2004; Zhou & Leydesdorff, 2006). Moreover, China is home to an extremely diverse primate community that includes 25–27 species from four subfamilies (Lorisinae, Cercopithecinae, Colobinae, Hylobatinae) and 7 genera (*Nycticebus*, *Macaca*, *Rhinopithecus*, *Semnopithecus*, *Trachypithecus*, *Hoolock* and *Nomascus*) (Estrada et al., 2017). These species differ markedly in body mass, social organization, mating systems, feeding ecology, and life history strategies. For example, group size among Chinese primates ranges from less than five to over 400 individuals (Qi et al., 2014). Similarly there are species described as solitary or inhabiting neighborhoods, whereas others live as socially monogamous pairs, one adult male – two adult female groups, harems or large one male multi-female breeding groups (OMUs), multimale- multifemale groups, and modular or multilevel societies composed of several OMUs plus an associated all male unit (Fan et al., 2010; Qi et al., 2014). Given this diversity, and the fact that studies of wild Chinese nonhuman primates have been conducted principally by in-country teams of scientists, Chinese primatologists are positioned to both empirically and theoretically advance the discipline of primatology in new and exciting ways. 

## THE CHALLENGES OF CONDUCTING PRIMATE FIELD STUDIES IN CHINA

### Long-term studies vs. multigroup comparisons

Despite the considerable challenges of conducting long-term field studies, several research teams in China have collected data on the same primate group or population for a period of 10–30 years (e.g., Tibetan macaques (*Macaca thibetana*), golden snub-nosed monkeys (*Rhinopithecus roxellana*), rhesus macaques (*Macaca mulatta*) black crested gibbons (*Nomascus concolor*), black-and-white snub-nosed monkeys (*Rhinopithecus bieti*), white-headed langurs (*Presbytis leucocephalus*)). This commitment has resulted in an extensive longitudinal database from which to distinguish and compare year-to-year variation in individual fertility, reproductive success, social interactions, and group dynamics with long-term patterns that may be indicative of important evolutionary processes. Other teams have studied multiple groups of the same species living in different habitats and can address critical research questions concerning which specific aspects of a species’ behavior and biology are conservative and remain constant across different environmental conditions and which traits are more plastic and vary (and to what degree they vary) under changing conditions (Zhao et al., 2011). The analysis and publication of these data will likely change current perspectives on many aspects of primate behavior, ecology, social systems, and mating strategies.

### Physiological challenges exploiting high altitude forests

A number of Chinese primates inhabit high altitude temperate forests (including gibbons, leaf-monkeys, macaques, and snub-nosed monkeys) characterized by relatively long, cold winters. Although primates living in both tropical and temperate regions face significant ecological challenges in locating resources and consuming a nutritionally balanced diet, temperate-living populations encounter additional problems associated with hypoxia and the increased energetic costs (or reduced energy expenditure) of remaining thermoneutral during cold daytime and nighttime temperatures. The set of behavioral, developmental, genetic, nutritional, and physiological factors that enable nonhuman primates to successfully exploit temperate habitats are poorly understood, and Chinese primatologists are poised to take the lead in developing new theoretical frameworks to examine concepts of adaptation and adaptability in primate evolution.

### Ecological data

Over the past decade, Chinese primatologists have increasingly published their research in high-impact journals (Fan & Ma, 2018). The majority of these studies have focused on primate social organization, social relationships, reproductive behavior, population genetics, and group size and composition. Only a limited number of studies have included a strong ecological component in the research. In part this relates to the fact that many primate species in China have extremely large home ranges (e.g., golden snub-nosed monkeys have home ranges of >10 km^2^) and presently are restricted in their distributions to mountainous regions and steeply sloped terrain unsuited to agricultural development. This makes the systematic collection of ecological data on tree species abundance, distribution, and resource productivity more difficult (unpublished data). In addition, several primate groups that have been the target of long-term studies are semi-provisioned to facilitate habituation. This complicates the ability of researchers to test theories examining interrelationships between day range, patterns of habitat utilization, diet, and nutritional strategies and should be minimized or eliminated. 

Overcoming these challenges to promote greater emphasis on the feeding ecology and foraging strategies of Chinese primates is needed to better understand the causative effects of food availability on group size, feeding competition, and reproductive success. In the absence of detailed ecological information, researchers are constrained to offer explanations of their behavioral results that are based on untested assumptions present in the primate literature. For example, although it is tempting to assume that golden snub-nosed monkeys living in high altitude forests are food-limited during the long winter season, reduced food availability is not consistent with data indicating that golden snub-nosed monkeys live in large, cohesive, multilevel societies of several hundred individuals during all months of the year. Moreover the assumption of reduced food availability seems to contradict the fact that female nutritional requirements increase during pregnancy and female golden snub-nosed monkeys are pregnant during the winter. Finally, all group members must increase food intake during the winter to offset the additional energetic demands required to remain thermoneutral. Thus, either food available to golden snub-nosed monkeys is not limiting during the winter, or individuals of this species have evolved a set of successful dietary, digestive, or behavioral strategies to overcome this challenge.

A stronger ecological focus would provide a constructive framework for identifying functional and adaptive relationships among primate behavior, anatomy, and ecology, as well as opening new avenues of inquiry that include species differences in spatial memory, decision-making, and cognition. Equally important, a stronger ecological focus will enable researchers to identify which species of trees and food items are most critical for conserving vulnerable primate populations. This would facilitate the success of projects involved in habitat restoration, the construction of forested corridors designed to facilitate migration and gene flow among isolated subpopulations, and habitat expansion into environmentally suitable and newly set aside protected areas. 

## PRIMATE CONSERVATION

At present some 80% of primate species in China are listed as vulnerable, endangered, or critically endangered by the IUCN (Estrada et al., 2017). We are at a historic moment in which China has the opportunity and the human capital of well-trained scientists to reverse decades of environmental degradation and biodiversity decline. Conservation must be a priority and a major component of all field and captive studies of Chinese primates. 

## PRIMATOLOGY AS A SCIENTIFIC DISCIPLINE

The rapid expansion of Primatology in China has been fueled by an exponential increase in the number of individuals’ receiving advanced degrees. Fan & Ma (2018) report that between 1984–2016 some 480 Chinese researchers received either a M.A. degree or Ph.D. in primatology. In recognition of this and other scientific achievements, in 2017 the International Primatological Society and the Chinese Government officially sanctioned the creation of the China Primatological Society (CPS). The CPS had its inaugural meeting in August 2017 in Xi’an, China. Some 200 scientists, directors of research institutes, students, and local governmental officials attended, including a small number of international primatologists from Australia, Canada, Japan, and the United States. Given the increased prominence of Chinese primatologists, the CPS is positioned to sponsor an official international, peer-reviewed, high-impact, scientific journal for the study of primate behavior, ecology, conservation, and evolution. 

It is clear that Chinese scientists have and will continue to play an increasingly important role in advancing the science of primatology in the coming decades. To facilitate this, I encourage research teams to expand their commitment to cross-species comparisons rather than each team focusing it efforts on only one or two taxa. Moreover, few research teams are currently studying the two extant loris species in China (*Nycticebus bengalensis* and *N. pygmaeus*), as well as several species of macaques (*Macaca munzala*, *M. leucogenys*, *M. leonia*, M. *arctoides*) and langurs (*Semnopithecus schistaceus*, *Trachypithecus crepusculus*, *T. pileatus*, and *T. phayrei*) (Fan & Ma 2018). Given the number of primatologists in China, field studies of these taxa, many of which are threatened, must be expanded. Finally, in order for Chinese primatologists to maximize their impact on the discipline, researchers should consider studying primates inhabiting other regions. There are some 504 species of primates worldwide (Estrada et al., 2017). Conducting research on species of primates from other parts of Asia, Africa, and the Neotropics will provide Chinese primatologists with the broadest and most informed comparative perspectives needed to rethink, refine, and test new theories in primatology.
